# Hospitals of London, Paris, Dublin and Edinburg

**Published:** 1840

**Authors:** 


HOSPITALS AND MEDICAL SCHOOLS OF LONDON, PARIS, DUBLIN AND EDINBURG.

Under this head we expect to be able to furnish our readers with a series of interesting sketches, from the pen of our friend, Dr. Moses L. Linton, one of the most gifted and promising young physicians in Kentucky, now on a tour of professional improvement in Europe. Dr. L. has industry, a noble ambition, a true love of science, and describes accurately and elegantly, whatever he sees. The two following letters present a sketch of some of the scientific institutions of London and Paris: Y.

London, December 5th, 1839.

*Dear Sir*:—I propose, during my stay in Europe, addressing to you a few letters, containing notices of the Hospitals, and various medical institutions of London, Paris, Dublin and other places distinguished for the cultivation of the profession; and without attempting, by a labored preface, to show that such notices either will or ought to be interesting to American physicians, I shall proceed directly to the subject.

*St. Bartholomew's Hospital*, the oldest establishment of the kind in London, is a capacious edifice of stone, occupying the site of the ancient priory of St. Bartholomew, and near the centre of the city proper. The principal entrance is through a large arch, in a rustic basement, over which stands the statue of Henry VIII. Above is an interrupted semi-circular pediment, on the segment of which recline two emblematical figures, representing lameness and sickness. The interior arrangements are convenient and ample—the professional attendance able, well divided and abundant—the nurses kind and numerous; and the greatest order appears to reign throughout the whole. This Hospital was founded by a monk, Rahere, in 1102, and refounded by Henry, after the destruction of the monasteries in the sixteenth century. It escaped the great conflagration of 1666, that destroyed five-sixths of the whole city, and a great portion of the suburbs.

Attached to this, as to most other similar institutions in the city, is a medical school, in which the various branches of the profession are taught, viz: Principles and Practice of medicine, by Doctors Latham and Burrows; Anatomy, Physiology and Pathology, by Edward Stanley, F. R. S.; Demonstration of practical Anatomy and superintendence of dissection, by Mr. Wormald; Chemistry, by Mr. Brande at the Royal Institution; Midwifery and diseases of women and children, by Dr. Rigby; Materia Medica, by Dr. Roupell; Surgery, by William Lawrence, F. R. S., and surgeon extraordinary to the Queen, &c. During the summer course, lectures are delivered on the collateral branches of Botany, Medical jurisprudence, &c. St. Bartholomew's contains five hundred and fifty beds, of which three hundred and seventy-five are allotted to surgical, and one hundred and seventy-five to medical cases. The in-patients amount annually to above five thousand, and the out-patients to above twenty-three thousand.

The fees paid by students are high, being from five to nine guineas to each lecturer, per winter session, thirty guineas for the privilege of attending the medical practice of the Hospital for an unlimited period. The privilege of attending the surgical practice is purchased by the payment of a still higher fee, being eighteen guineas for six months. The museum of Anatomy contains five hundred and forty specimens of natural human structure; seventeen hundred of morbid human structure; three hundred and ninety of the form and structure of parts in animals; one hundred and fifty of congenital defects in man and animals; one hundred and ninety casts and models in wax and plaster of Paris. The library contains upwards of four thousand volumes. Strangers and foreigners belonging to the profession, who visit this Hospital, are treated with the utmost courtesy, and freely admitted from day to day into all its wards, lecture-rooms. and other places of interest. The same may be said of all the Hospitals in London.

In the management of diseases, both medical and surgical, I saw and heard but little that is new, or worthy of especial notice. Syphilis is treated by the various preparations of mercury internally administered, or externally applied, or both. As a local application, the black wash is in high repute—as a diet-drink, sarsaparilla. When, however, the disease seems not to yield, or to grow worse, as is often the fact, under the mercurial treatment, the hydriodate of potash is substituted in its stead, and Mr. Stanley, one of the surgeons, informs me, that, given in doses of from five to eight grains, thrice daily, he has used the article in such cases with decided advantage.

The great attraction which this institution affords alike to the transient visitor and the regular student, is Mr. Lawrence, whose varied erudition as a scholar and medical philosopher, eloquence and perspicuity as a lecturer, and tact as a surgical operator, few have had the good fortune to surpass. I have been listening to him for some evenings past, on the subject of *tumours*, and I certainly never heard it discussed more ably. According to his views, the following are the diagnostic traits by which *malignant*, may be distinguished from *simple* tumours.

1st: The former are generally deep-seated; whereas the latter are superficial.

2nd: The growth of the former is rapid—of the latter, slow.

3rd: The former are generally painful, whilst the latter produce inconvenience only by their size; and

4th: The latter produce no constitutional disturbance, whilst the former are attended by emaciation, hectic fever, and various other evidences of vital derangement. It is not important, he remarked, to know the composition of a tumour previously to exsecting it. You must, in most cases, exsect it before you *can* know its constitution. He observed that it is almost impossible to classify them, and certainly impossible to refer many that are met with to any of the classifications of authors, their texture and composition being as various as the combinations into which animal elements, under the influence of vital action, can enter. Before determining on an operation, said he, the following facts ought to appear :

1st : That the tumour is causing great inconvenience, pain, or disturbance of some kind.

2nd : That the operation is safe, or at least promises to increase the chances of life ; and

3rd : That the patient, after being apprized of all the circumstances, desires it.

It is a law of tumours, he said, that they are disposed to resemble in structure, the parts in the neighborhood of which they are formed : thus the fatty tumour is found in the adipose layer that envelopes the body ; the glandiform tumour, called the pancreatic, under the jaw, in the region of the salivary glands, &c., &c.

This view of the subject was, I confess, in some respects, new and interesting to me. It is certainly a simplification of a very *abstruse and knotty* department of surgery.

As an operator, the reputation of Mr. Lawrence is truly enviable. Amongst other operations, on Saturday last I saw him perform one for the relief of Trichiasis. The eye was very much inflamed, and entirely incapable of performing its function. By means of a needle he passed a thread through the lid near its margin, by which he held it far enough from the ball, to cut away with safety as much of that margin, as included the bulbs if the ciliæ. He remarked that the operation was always followed by immediate relief, and, when the inflammation had not proceeded to serious structural lesion, a rapid restoration to the enjoyment of vision.

---

PARIS, March 25, 1840.

*Dear Sir*:—As what I have said in a former letter in relation to St. Bartholomew's Hospital, applies in a great degree, to all similar institutions in London, I shall pass without further notice of them, to a brief account of medicine in Paris—a subject to which I propose devoting this and a few succeeding letters; and as the University of France includes, presides over, governs, and directs the teaching, not only of this, but every other branch and grade of science in the kingdom, a sketch of the outlines of its organization, will, I conceive, form an appropriate preface.

At the commencement of the revolution there existed in the various Departments ten or twelve Universities, besides various Colleges and Schools, founded and sustained by the different religious orders. At that period, the whole were dissolved, and one imperial University established for all France; and placed under the direction of a Grand Master and Council. M. Cousin is at present Grand Master, or Minister of public instruction. The council royal, of which he is the president, consists of seven members. This body may be considered the supreme tribunal, not only of the University, but of the many bodies or associations of learned men, in which the kingdom, but more especially the capital, abounds; and at the head of which may be placed the Institute.

The University consists of, or is divided into about twenty-five academies, situated in the principal cities of the kingdom, and having jurisdiction over their respective sub-divisions. Each of these academies has its particular council or government—its primary, secondary, and superior instruction; or in other words, its primary schools, its colleges and its Faculties.

The organization of the University is indeed strikingly analogous to that of the government of the United States—each academy, as each state, having its particular laws and regulations, though subject in a great degree to a common head, or central power.

It should be remarked however that there are but three of these academies possessing medical faculties empowered to confer the doctorate, viz: those of Paris, Strasbourgh and Montpelier. Most of them have *secondary* medical schools, and many of them Faculties of Theology and Law. It will thus be seen that the University is colossal and grasping, that from the highest Faculties, down to the boarding-school where the first elements are taught, nothing escapes its attention or eludes its vigilance—a pyramid of intellect and science, founded and reared by the giant genius of Napoleon.

Having thus glanced at the University in general, we come now to a somewhat particular notice of its most renowned and important branch—the Academy of Paris.

Besides its numerous elementary schools for primary instruction, and its many royal and communal colleges for the higher branches of education, this Academy contains the following Faculties:

1st. Of *Theology.*—Comprising courses on the doctrines and evidences of christianity, sacred history, church government, dogmatic theology and sacred eloquence.

2d. Of *Letters.*—This Faculty gives lectures on Greek literature and philosophy, ancient and modern, Latin and French eloquence and poetry, French history and literature, geography and foreign literature.

3d. Of *Sciences.*—Here are courses on algebra, astronomy, mechanics, natural philosophy, chemistry, mineralogy, botany, zoology, descriptive geometry, comparative zoology and physiology, and geology.

4th. Of *Law.*—In this school, there are lectures on the Institutes of Justinian, the civil code, the pandects, the laws of nations, the history of law, the constitutional law of France, &c.

5th. Of *Medicine.*—This Faculty is composed of twenty-six professors besides a great many agrégés or adjuncts. The number of chairs or courses is eighteen, as will be seen by consulting the following table:

*Anatomy*, Breschet.—*Pathological Anatomy*, Cruveilhier.—*Medical Chemistry*, Orfila.—*Pharmacy and Organic Chemistry*, Dumas.—*Hygiene*, Royer Collard.—*Surgical operations and apparatus*, (on the *Cadaver*,) Richerand.—*Surgical Pathology*, Dumeril and Piory.—*General Pathology and Theraputics*, Andral.—*Materia Medica and Therapeutics*, Berard.—*Medical Jurisprudence*, Adelon.—*Physique Medicale*, Pelletan.—*Accouchements and diseases of Women and Children*, Moreau.—*Histoire Naturelle Medicale*, Richard.

Three clinical courses given at the Hospital as follows:

*Cliniques Chirurgicales.*—Roux, at Hotel Dieu; Velpeau, at La Charitie; Sanson, at La Pitie; Cloquet, at the Hospital of the Faculty.

*Cliniques Medicales.*—Fouquier and Bouillaud, at La Charitie; Ch mel, at Hotel Dieu; Rostan, at the Hospital of the Faculty.

*Cliniques d'Accouchements.*—Paul Dubois, at the Hospital of the Faculty.

A part of these courses is given in the winter, and a part in the summer session, thus constituting two terms, per annum, each of five months duration. The lectures are delivered at the Ecole de Medicine, with the exception of the cliniques.

A word in relation to this magnificent building. Its first stone was laid by Louis the XV., in 1769. The front towards the street consists of a colonnade of the Ionic order, one hundred and ninety-eight feet in length, supporting an entablature and attic. Above the principal entrance, is a bas-relief, representing Louis XV, accompanied by Wisdom and Beneficence, and a Genius with a plan of the school. The court or area which the building encloses, is ninety-six feet in length, and sixty-six in breadth. The inner, or principal front, presents a projecting mass, composed of six large Corinthian columns, surmounted by a triangular pediment, on which is a bas-relief, allegorical of the theory and practice of medicine. On the wall are medallions, containing portraits of the celebrated Surgeons, Pétard, Paré, Petit, Mareschal and Peyronie.

The principal amphitheatre will contain between one thousand and fifteen hundred persons. It possesses three fresco paintings by Gibelin; one represents Esculapius teaching the elements of Medicine; in another the Surgeons are dressing the wounded after battle; and in the third, the Genius of Medicine crowned by Fame, is bestowing prizes upon pupils and academicians.

In the salle d'assemblée is a painting by Gerodet, representing Hippocrates refusing the presents of the king of Persia to practice his art amongst the enemies of his country. It is surrounded by the busts of the most famous French Anatomists, Physicians and Surgeons. One of the saloons of this building is appropriated to the library, containing more than thirty-five thousand volumes; and five others to the museum of Faculty, consisting of an extensive collection of anatomical preparations, normal and abnormal; an arsenal of surgical instruments, objects of natural history, specimens of the materia medica, and chemical and physical apparatus.

About one hundred yards from this edifice is a series of buildings in which the agrégés of the Faculty give lectures on anatomy, and operative surgery, and in which are a great many rooms for dissections. This establishment is known by the name of the Ecole Pratique. Near the Jardin des Plantes is another still more extensive, appropriated to anatomical studies. To this place, "*Clamart*," the carts, employed for the purpose, bring every morning the unclaimed dead of the various hospitals. When they arrive, and the bodies are deposited in a central hall, a bell is rung to summon the students to what would appear to the uninitiated a melancholy market. It is scarcely necessary to mention that the friends of the dead, if friends they have, can claim their bodies at the hospital, and afford them that last gift of charity—a grave.

I shall close this *coup d'œil* of medical and other institutions of learning in France, by a short account of the Museum of Dupuytren. This distinguished Surgeon having left by his will two hundred thousand francs for the creation of a chair of Pathological Anatomy, M. Orfila profited by the circumstance in soliciting the Royal Council of public instruction, of which he is a member, to establish a Museum exclusively devoted to Pathological Anatomy—the request was granted, and gratitude to the generous founder of the new chair gave it a name.

This Museum occupies a vast saloon in which elegance and taste are associated with all the conveniences of its destination. It contains the principal pathological alterations, virieties of conformation, and anomalies of organization to which the human system is liable. All these are grouped or classified in a manner that renders their inspection easy, as well as interesting and instructive.

The subjects noticed in this letter, though rather dry and statistical, will not, I hope, prove entirely uninteresting. In my next I shall endeavor to afford something of a more practical character.

